# Do we pay enough attention to science in medical education?

**Published:** 2018-07-27

**Authors:** W. Wayne Weston

**Affiliations:** 1 Schulich School of Medicine and Dentistry, Western University, Ontario, Canada

“The need for a fundamental redesign of medical training is clear.”^1^

What is the proper place for the basic medical sciences in the medical school curriculum? The famous Flexner Report of 1910, oft-quoted, less often read, highlighted this question which has remained a cause for concern and debate ever since as demonstrated by the article by Coderre, Ripstein, Veale and McLauglin in this issue of the CMEJ.^2^ In this commentary I will survey some of the key developments in basic science teaching over the past 100 years and then address some important recent developments in the science of medical education. It is perhaps by building on the learning sciences that we have the best opportunities to address the recommendation of the Future of Medical Education in Canada Project to “Build on the Scientific Basis of Medicine.”^3^

Flexner argued that a four-year curriculum be adopted with the first two years devoted to the basic medical sciences which he defined as anatomy (including histology and embryology), physiology (including biochemistry), pharmacology, pathology, and bacteriology. He felt that the more fundamental sciences of biology, chemistry, and physics should be studied before medical school. He recommended that medical schools be brought into the university and that teaching methods involve fewer lectures, greater use of the laboratory, and more contact with patients. Although we have adopted most of these recommendations, we continue to make modifications in the subject matter of the curriculum and in how we teach. The number of sciences taught has been expanded to include the behavioural and social sciences, and several other important topics have been added to the first two years of the curriculum such as ethics and professionalism, clinical skills including communication, quality improvement, indigenous issues, and the humanities.^4^ This leaves less time for teaching the traditional basic sciences. In addition, curriculum planners recognized that an overcrowded timetable results in less learning^5^ and that passive methods of learning, such as lectures, are inferior to other forms of teaching in producing long-term retention of knowledge.^6,7^ Based on the writings of John Dewey, Flexner recommended active methods of learning.^8^ To illustrate this pedagogic approach, Flexner quotes Cabot and Locke: “If one had one hundred hours to learn to ride a horse or to speak in public, one might profitably spend perhaps an hour (in divided doses) in being told how to do it, four hours on watching a teacher do it, and the remaining ninety-five hours in practice, at first with close supervision, later under general oversight.”^9^

As a result of these insights, hours of basic science teaching were cut and teaching methods were changed to encourage active learning by students. Some have expressed concern that a lessened focus on the basic sciences diminishes the professional status of medicine as a learned career and that the quality of medical care is reduced.^10^ Others remind us that medical care is about so much more than applied basic science.^11-13^ The role of the physician is to attend to the many ways that a patient’s disease interferes with their ability to achieve what really matters to them – “to live their chosen lives.”^14^ This requires a deep understanding of the human condition and the ability to connect to patients with care and compassion. It demands skill in using the sciences in making an accurate diagnosis and in formulating options for treatment as well as skill in communication in order to obtain an accurate story of the patient’s illness and to ascertain their values and preferences regarding treatment. This is a tall order. It can only be started during medical school but is never finished.

Is there too much to learn in the time available? Have the basic sciences been shortchanged? Do we need to make medical education longer? There is unlikely to be any appetite for a longer curriculum. In fact, in an online survey in 2014, 47% of education deans in the U.S. agreed that there is a need to shorten medical school and over one-third of medical schools were considering the development of a three-year program.^15^ In Canada, McMaster and Calgary have had three-year undergraduate medical curricula for many years. But the shortening of training is achieved primarily by eliminating breaks and limiting summer vacation with the result that the total length of the 3-year curriculum is roughly the same as in 4-year programs. If lengthening medical school is not the answer to the over-crowded curriculum, is there another way to make sure graduates of medical school are well-versed in the basic sciences without eliminating all the new topics considered necessary for the preparation of a well-educated physician? The answer might be found in another group of sciences – the learning sciences.^16-19^

Serious study of medical education began in the early 1950s at the University of Buffalo School of Medicine. Teachers at the medical school began meeting with colleagues from the School of Education to develop the Project in Medical Education to learn how to apply what was known about education to improve teaching and learning in medical school.^20^ Since then, educators have learned a great deal about how people learn that would be useful in guiding the development of curricula and informing teachers about how they can be more effective. The publication of How People Learn^21^ in 1999 was a watershed that summarised over 30 years of work by learning scientists and made it accessible to all educators. Since then, the learning sciences have produced much valuable additional research.^22-25^ Some argue that we have entered the age of evidence-based education.^26-28^ In the inaugural issue of Best Evidence Medical Education (BEME) Guide Number 1 in 1999, the authors argue that: “The adoption of best evidence medical education does not require the teacher to be a researcher in education. It does require the teacher to be able to appraise the evidence available and come to a decision on the basis of his or her clinical judgement.”^29^ An article by Cutting and Saks provides twelve tips for utilizing what we know about learning to improve medical education.^30^

Students forget much of the basic science they learned in the first two years of medical school.^31,32^ In one study of retention of anatomy in a traditional curriculum, the curve for forgetting was the same as that for nonsense syllables.^33^ This can be explained by the failure of medical schools to consider what is known about human learning in designing curricula. In 1978, Stephen Abrahamson, having visited half of all the medical schools in the U.S. over a period of twenty years, described nine “diseases of the curriculum.” Since then, research in the cognitive sciences has provided a better understanding of how these *pathologies* interfere with student learning. One example is curriculum hypertrophy or *curriculomegaly* based on the idea that “new knowledge deserves to be recognized and included, but what we have always done cannot be tampered with.”^34^ Cognitive load theory, based on the limited capacity of working memory to attend to and encode information when there are real time demands, explains why students learn less when lectures contain too much information.^35^ Organizing a lecture into bite-sized chunks, with breaks between them, will reduce the load. Forgetting is primarily a problem of recall. Relearning and rehearsal at expanding intervals and frequent testing have all been shown to improve long-term retention.^36^ Deep learning, i.e., making it meaningful by connecting the new learning to what we already know, takes time. But when we are presented with too many new ideas to reflect on, we resort to surface learning – skimming to get the gist of the ideas. The more deeply we process new data, the more connections we form with other knowledge, which serve as retrieval cues when we need to call up the information later. That explains why surface learning, which generates few retrieval cues, is often forgotten. For example, in a study of how medical students learn anatomy, those who combined memorization, visualization, and a focus on understanding performed significantly better on final exams than those who used only memorization.^37^

One of the most important ways to improve recall of basic science learning in the clinical setting is to integrate basic science concepts with clinical content.^38-41^ That way, the clinical issues will form retrieval cues to the basic science material. But it’s not that simple. When a basic science concept is learned in the context of a clinical scenario, it may be remembered only in the context of a very similar scenario. Some research on Problem-based Learning shows that students perform worse than students in a traditional medical curriculum.^42^ Specific – sometimes trivial - features of the scenario become retrieval cues rather than the underlying deep structure of the clinical problem. But using multiple examples and explicitly identifying the underlying concepts significantly improved recall.^43^ Flexner recognized the importance of teaching the basic sciences using methods that demonstrated their clinical relevance:

…undergraduate [medical] instruction will be throughout explicitly conscious of its professional end and aim. In no other way can all the sciences belonging to the medical curriculum be thoroughly kneaded. An active apperceptive relation must be established and maintained between laboratory and clinical experience. Such a relation cannot be one-sided; it will not spontaneously set itself up in the last two years if it is deliberately suppressed in the first two There is no cement like interest, no stimulus like the hint of a coming practical application.^44^

But, it has proved challenging to do this.^45^ Many basic science teachers have not had a medical education, so it is not easy for them to know what aspects of their courses are most relevant for future physicians nor is it easy to think of clinical examples to illustrate basic science concepts. Similarly, clinical educators have often forgotten much of the basic science they learned as students. Ideally there should be opportunities for teachers from the basic sciences and the clinical disciplines to collaborate on designing and teaching the curriculum. But, with all the other demands on their time and lack of academic reward for such efforts, such collaboration is hindered. Another approach to integration is a return to basic science courses throughout the clinical years of medical school.^46,47^ At that point in their education, students are more aware of the relevance of the basic sciences. An additional challenge for integration is finding a way to select which of the huge number of basic science concepts to keep in the curriculum. Goldszmidt et al. compared one group of students given instruction on the basic physics of lung sounds with a control group that were given instruction similar to standard textbook descriptions of the physical examination of the respiratory system. Students provided with instruction on the acoustics of lung sounds performed significantly better on questions related to the interpretation of physical exam findings. Based on this, the authors suggest, “…a decision to include certain basic science teachings into the curriculum should take into account their ability to support causal knowledge learning.”^48^ Klement et al. provide a useful outline of strategies for associating basic science concepts with a medical application or disease.^49^

It is important to remember that, education, like medicine, is more than applied science. Good education requires creative artistry and craft skills. “…teaching is an art in the sense that teachers, like painters, composers, actresses, and dancers, make judgments based largely on qualities that unfold during the course of action.”^50^ Although teachers need routines and practices, based on best evidence of their effectiveness, they must not be so dominated by them that they cannot respond creatively to contingencies that are unexpected. As Kenneth Eble advocates: “Though I would have teachers systematically pursue teaching skills, I would also have them recognize an essential looseness in their craft. Teaching, like writing, is a craft that finds its exact way each time out.”^51^ Contrary to a popular myth about Flexner, he favoured the inclusion of the humanities in medical education. “In later years, Flexner felt that the medical course had become overwhelmed with science to the exclusion of the humanistic aspect of medicine.”^52^ He wrote in 1925, “Scientific medicine in America – young, vigorous and positivistic – is today sadly deficient in cultural and philosophical background.”^53^

An encouraging development, that has the potential to improve medical education more than perhaps any other intervention, is the increasing and worldwide development of masters-level programs in health professions education.^54^ Until 1996, there were only seven such programs in the world; in 2012 there were 78; in 2017, 127. The Foundation for Advancement of International Medical Education and Research keeps an updated list on its website at https://www.faimer.org/resources/mastersmeded.html. Such programs give those interested in focusing their careers in education some of the skills they need to lead educational developments in their schools and the credentials it supplies assists in their career advancement. A related development is the creation of Departments, Units, and Centres for educational research in many medical schools that build a critical mass of interprofessional educational researchers who teach and learn from each other to support the scholarship of education that deepens our theoretical understanding of medical education and provides useful theory and evidence of effectiveness to guide our curriculum planning and day-to-day teaching.^55,56^

Since Flexner’s report on medical education over 100 years ago recommended better education in the basic sciences, medical schools have reduced the curriculum time for basic science courses to make way for other important topics and to reduce curriculum overload. To ensure this does not weaken our students’ understanding of the basic sciences, we need to change both what we teach and the way we teach. Studies of how people learn together with research in medical education are providing the knowledge we need to create curricula informed by evidence in education and what we have discovered about human learning. If basic science and clinical teachers learn from the educational scholars and researchers and if we align academic rewards for faculty with the needs for improved medical education, we can create curricula that Flexner himself would have been proud of.
